# World Health Organization Enhanced Gonococcal Antimicrobial Surveillance Programme, Cambodia, 2023

**DOI:** 10.3201/eid3007.240354

**Published:** 2024-07

**Authors:** Vichea Ouk, Heng Lon Say, Mot Virak, Serakea Deng, Rebekah Frankson, Robert McDonald, Ellen N. Kersh, Teodora Wi, Ismael Maatouk, Sebastiaan van Hal, Monica M. Lahra, for the EGASP Cambodia working group

**Affiliations:** National Center for HIV/AIDS, Dermatology and Sexually Transmitted Diseases, Phnom Penh, Cambodia (V. Ouk, H.L. Say);; Laboratory of the National Institute of Public Health, Phnom Penh (M. Virak);; World Health Organization Office of Cambodia, Phnom Penh (S. Deng);; Centers for Disease Control and Prevention, Atlanta, Georgia, USA (R. Frankson, R. McDonald, E.N. Kersh);; World Health Organization, Geneva, Switzerland (T. Wi, I. Maatouk);; The University of Sydney, Sydney, New South Wales, Australia (S.J. van Hal);; The Prince of Wales Hospital, Randwick, New South Wales, Australia (M.M. Lahra);; The University of New South Wales, Sydney (M.M. Lahra)

**Keywords:** Antimicrobial resistance, Neisseria gonorrhoeae, surveillance, sexually transmitted infections, World Health Organization Enhanced Gonococcal Antimicrobial Surveillance Programme, Cambodia, bacteria

## Abstract

To determine antimicrobial susceptibility of *Neisseria gonorrhoeae*, we analyzed phenotypes and genomes of 72 isolates collected in Cambodia in 2023. Of those, 9/72 (12.5%) were extensively drug resistant, a 3-fold increase from 2022. Genomic analysis confirmed expansion of newly emerging resistant clones and ongoing resistance emergence across new phylogenetic backbones.

The World Health Organization (WHO) Enhanced Gonococcal Antimicrobial Surveillance Programme (EGASP) was established in Cambodia in 2020. The program is led by the National Center for HIV/AIDS, Dermatology and Sexually Transmitted Diseases, Cambodia, in partnership with the WHO headquarters, regional and country offices, and WHO Collaborating Centres based in the US Centers for Disease Control and Prevention (Atlanta, GA, USA) and New South Wales Health Pathology (Sydney, NSW, Australia). The WHO Collaborating Centre for Sexually Transmitted Infections and Antimicrobial Resistance in Australia provides technical support for the surveillance, including whole-genome sequencing of isolates. As part of the EGASP protocol, *Neisseria gonorrhoeae* isolates were collected from men with urethral discharge seen at 6 sentinel clinics in Phnom Penh and 4 sentinel clinics in neighboring provinces. EGASP protocols are set to alert public health authorities of strains approaching resistance. EGASP sets *N. gonorrhoeae* MIC alerts for ceftriaxone, cefixime, gentamicin, and azithromycin (*1*) (Table).

**Table:** Antimicrobial susceptibility of *Neisseria gonorrhoeae* isolates from the World Health Organization EGASP, Cambodia, 2022–2023*

​Antimicrobial drug	​​2022, n = 76, no. (%) (*2*)	​2023, n = 72, no. (%)
​Ceftriaxone >0.125 μg/mL†	​29 (38)	​22 (31)
​Cefixime >0.25 μg/mL†	​29 (38)	​22 (31)
​Azithromycin >2.0 μg/mL†	​0	​1 (1.4)
Azithromycin >256 μg/mL†	​3 (4)	​9 (12.5)
​Ciprofloxacin resistant >1 μg/mL‡	​74 (97)	​70 (97)
​Penicillin resistant >2 μg/mL‡	71 (93)	59 (82)
​Spectinomycin resistant >128 μg/mL‡	​0	​0

*EGASP, Enhanced Gonococcal Antimicrobial Surveillance Programme. †The Clinical Laboratory Standards Institute criteria for resistance to ceftriaxone, cefixime, and azithromycin have not been established. The EGASP MIC alert value criteria were ceftriaxone, MIC >0.125 μg/mL; cefixime, MIC >0.25 μg/mL; azithromycin, MIC >2.0 μg/mL; and azithromycin, MIC >256 μg/mL. ‡Antimicrobial susceptibility criteria established by the Clinical Laboratory Standards Institute (https://www.clsi.org) were used to determine resistance to the antibiotics tested in the EGASP for ciprofloxacin, penicillin, and spectinomycin.

The first report from EGASP (*2*) detailed findings from 76 *N. gonorrhoeae* isolates collected by EGASP in Cambodia in 2022 and tested at the WHO Collaborating Centre for Sexually Transmitted Infections (STIs) and Antimicrobial Resistance in Australia. Of those, 29/76 (38%) isolates had alert level MICs for ceftriaxone (MIC >0.125 μg/mL) and cefixime (MIC >0.25 μg/mL) (*1*) and were also resistant to penicillin and ciprofloxacin. Almost all isolates harbored the mosaic *penA*-60.001 allele (n = 27) across 9 multilocus sequence types (MLSTs), determined by in silico typing. Those findings suggested that *penA*-60.001 allele carriage is more extensive than previously reported and indicated that widespread dissemination across the region might have already occurred (*2*). Furthermore, 3 *penA-*60*.*001–containing isolates (3/76; 4%) had an extensively drug resistant (XDR) phenotype (*3*), all from a single sequence type (ST), ST-16406, and similar to previously reported XDR *N. gonorrhoeae* isolates with links to the region (*3*).

We present data from an additional 72 gonococcal isolates collected in 2023 by the Cambodia EGASP team and referred to the WHO Collaborating Centre in Australia for phenotypic and genomic analysis. All 72 isolates underwent antimicrobial susceptibility testing. EGASP alert MICs for ceftriaxone and cefixime were detected in 22/72 (31%), a proportion similar to that reported in 2022 (38%) (*2*) (Table). In 2023, a total of 9 (12.5%) isolates had the XDR phenotype and the same MLST, ST-16406, a 3-fold increase from 4% of isolates with the same phenotype reported in 2022 (3/76). The percentage of isolates with resistance or elevated MICs to ciprofloxacin and penicillin remained high (82%–97%, Table).

Genomic analysis (*2*) showed that, of the 54 isolates with ceftriaxone alert MICs in 2022 and 2023, a total of 50 harbored the *penA*-60.001 allele and 3 harbored the recently described mosaic *penA-*273.001 allele (*4*). The remaining isolate harbored a novel allele classified as *penA-*60.003, which differed from *penA*-60.001 by a single-nucleotide polymorphism. Compared with 2022, the 2023 *penA*-60.001 allele was seen across 5 similar MLSTs (ST-1587, ST-7363, ST-8130, ST-11368, and ST-16406) and 3 other MLSTs (ST-7827, ST-14950, novel ST). Critically, across the 2 years of the EGASP study, sequences from only 8 isolates from Cambodia clustered with the widely disseminated FC428 clone (*5*), confirming both expansion of newly emerging resistant clones and ongoing emerging resistance across new phylogenetic backbones (Figure). Moreover, all *N. gonorrhoeae* isolates with azithromycin MICs >256 μg/mL were found to possess the 23S rRNA gene mutation (A2509G). All 12 isolates with an XDR phenotype detected during 2022–2023 belonged to a single ST (ST-16406), recently reported in Europe and the United Kingdom (*3*). Sequence reads are available from the National Center for Biotechnology Information (BioProject no. PRJNA909328).

**Figure:**
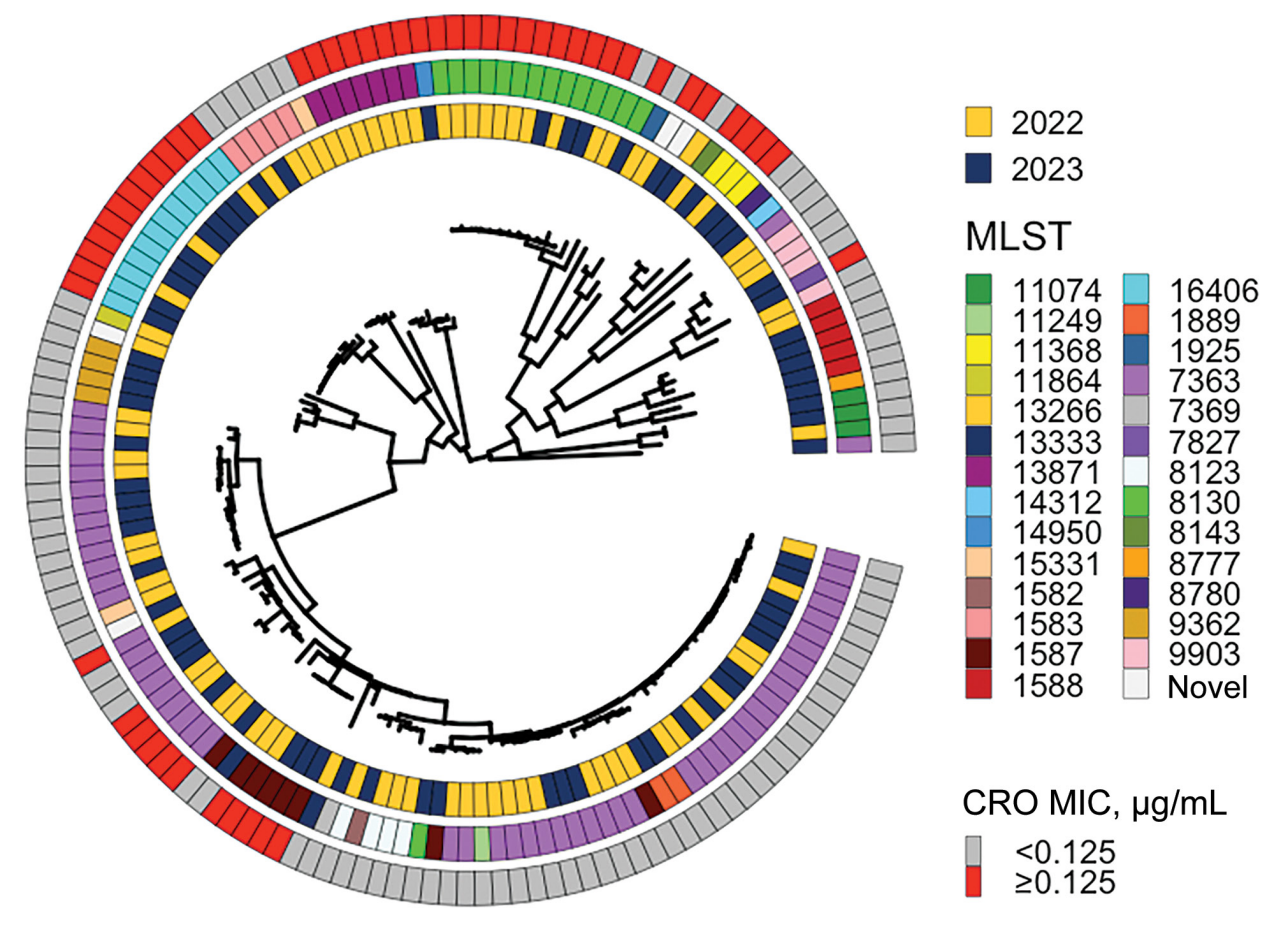
Circular midpoint rooted phylogeny of sequences from *Neisseria gonorrhoeae* isolates collected by the World Health Organization Enhanced Gonococcal Antimicrobial Surveillance Programme, Cambodia, 2022–2023. Associated metadata, year of isolation, MLST, and presence or absence of elevated MICs for CRO are depicted in concentric rings. White cells in the 3 rings correspond with the tree tip belonging to isolate FC428, the first *penA*-60.001-containing ceftriaxone-resistant isolate documented in Japan in 2015 (*6*). CRO, ceftriaxone; MLST, multilocus sequence type.

Our data provide further evidence for sustained transmission of *N. gonorrhoeae* strains with elevated MICs for ceftriaxone and increased expansion of isolates with elevated MICs to ceftriaxone and azithromycin that genomically cluster with the XDR *N. gonorrhoeae* phenotype. Furthermore, strains with elevated MICs for ceftriaxone continue to emerge across different phylogenetic backbones separate from the previously described FC428 clone, confirming concerns that biological fitness is not compromised by that allele (*5*) and consequently poses a substantial threat for gonococcal disease control.

The proportion of alert-level isolates detected across the 2 years (2022–2023) of WHO EGASP Cambodia is of considerable concern, given the implications for future empirical therapy. Moreover, 2 recent publications from China report rates of *N. gonorrhoeae* with ceftriaxone MICs >0.125 μg/mL at or beyond 9%, most associated with the mosaic *penA*-60.001 allele (*7.8*), suggesting a far wider regional problem. That gonococcal antimicrobial resistance surveillance across the Asia–Pacific region is largely unmapped (*9*) reinforces high-level concerns regarding this priority pathogen. There is an urgent need to prioritize surveillance and data reporting to inform local and regional disease prevention and control strategies.
